# Impact of General Anesthetics on Postoperative Infections—A Narrative Review

**DOI:** 10.3390/life15111662

**Published:** 2025-10-23

**Authors:** Taylor P. L. Butt, Lynn Jazzar, Palak Watts, Christian Lehmann

**Affiliations:** Department of Anesthesia, Pain Management and Perioperative Medicine, Dalhousie University, Halifax, NS B2H 0A3, Canada; taylor.butt@dal.ca (T.P.L.B.); ly778351@dal.ca (L.J.); palak.watts@dal.ca (P.W.)

**Keywords:** postoperative infection, immune modulation, anesthetic agents

## Abstract

Postoperative infections represent the most frequent complication after surgery. Anesthetic agents, while essential during surgical procedures to ensure unconsciousness, are becoming increasingly recognized as modulators of immune function. Volatile anesthetics have been identified as being able to attenuate the inflammatory response in diverse experimental models. Propofol, a widely used intravenous anesthetic, has also been described to exhibit strong anti-inflammatory mechanisms. This review synthesizes current cellular, experimental, and clinical evidence on the immunomodulatory effects of anesthetic agents, highlighting their impact on host defense mechanisms and postoperative infections. By exploring mechanistic properties and clinical outcomes, it underscores the importance of anesthetic choice in enhancing immune function and postoperative recovery.

## 1. Introduction

Postoperative infections such as surgical site infections (SSIs), pneumonia, and sepsis are among the most severe complications following surgical procedures. SSIs account for upwards of 20% of all healthcare-associated infections (HAI) and is reported to have an annual cost of $3.3 to $10 billion dollars [1]. Postoperative infections not only prolong hospital stay and increase readmission rates but also contribute to longer wound healing periods and increased mortality [2]. Despite recent improvements in surgical techniques and infection control protocols, postoperative infections remain a significant burden across surgical populations [3].

General anesthetics are used to induce loss of sensation or consciousness so that medical procedures can be performed in the absence of discomfort and pain. Anesthetics are increasingly recognized for their off-target biological activities, including immune modulation [4]. Several preclinical studies have shown that anesthetic agents have the ability to alter intra- and intercellular signaling pathways involved in immune cell function [5]. While these immunomodulatory effects may help to prevent excessive inflammation, they also raise concern about potential immune suppression, especially in the postoperative setting, a critical period for antimicrobial host response to infections [6]. As a consequence of immune suppression, patients may become more susceptible to postoperative infections, which can have significant effects on recovery.

Thus, anesthetic selection that can balance the immune response is critical. Anesthetics vary in their inflammatory immunomodulatory effects, where some may prevent excessive inflammation to further reduce tissue damage, while others may impair the immune response, resulting in increasing infection risks [4,7,8,9]. Therefore, understanding the specific immunomodulatory mechanisms and effects of each anesthetic is crucial for selecting the best agent to minimize infection risk and facilitate recovery.

**Figure 1:**
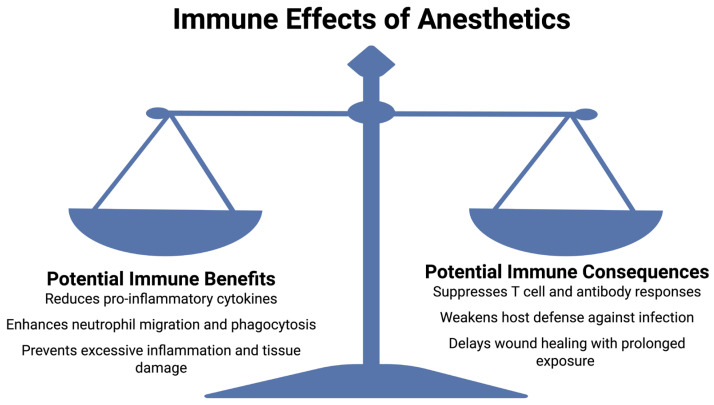
A summary of the immunomodulatory effects of anesthetic agents. Some anesthetics may provide immunological benefits by reducing pro-inflammatory cytokine release, enhancing neutrophil activity, and preventing excessive inflammation and tissue damage. However, they may also have detrimental immune consequences, including suppression of T cell and antibody responses, weakening of host defense, and delayed wound healing, particularly with prolonged exposure. This balance highlights the importance of anesthetic choice in modulation of postoperative immune responses. Created with BioRender.com.

This review aims to examine the immunomodulatory effects of commonly used anesthetics, such as propofol, sevoflurane, and isoflurane. By exploring both, in vitro and in vivo studies, as well as clinical evidence, we aim to understand how these anesthetics influence immune function and postoperative infection rates. Gaining a better understanding of these effects is crucial in the practice of anesthesia to better balance sedative and analgesic effects with infection prevention to ensure the best patient outcomes. The immunomodulatory effects of anesthetics are illustrated in Figure 1, providing a brief summary of the potential benefits and detrimental effects these anesthetics may have on the immune system.

## 2. Methods

This manuscript was prepared as a narrative review. A literature search was conducted to identify studies examining the immunomodulatory effects of anesthetics and their relationship to postoperative infection outcomes. The search was performed using PubMed, using the following MeSH terms: “surgical wound infection,” “immunomodulation,” “propofol,” “isoflurane,” “halothane,” “desflurane,” and “sevoflurane.” Studies were included if they were original research articles or reviews published in English, investigated the effects of general anesthetics on the immune system and postoperative infection, and used in vitro, in vivo, or clinical trial models. Specifically, observational pre-clinical studies, cohort studies and randomized controlled trials were included. Exclusion criteria included studies not assessing immune responses or infection-related outcomes, and articles without full-text access.

## 3. In Vitro Evidence

Understanding how anesthetic agents influence the immune system at the cellular level is essential for improving perioperative care and minimizing operative complications, including infections. While clinical studies provide insights into patient outcomes, in vitro experiments offer a controlled environment to analyze the direct molecular and cellular effects of anesthetics on immune cells. These studies allow researchers to evaluate changes in cytokine production, cell surface receptor expression, intracellular signaling pathways, and functional responses such as phagocytosis, antigen presentation, and T-cell activation. By isolating immune cells and exposing them to anesthetics in a dose- and time-dependent manner, investigators can uncover specific mechanisms by which these drugs either suppress or enhance immune function—effects that may not be readily apparent in vivo due to the complexity of whole-body interactions. In vitro data is particularly valuable in highlighting early immunological alterations that may contribute to postoperative immune suppression or impaired pathogen clearance. This section examines the in vitro immunomodulatory effects of five commonly studied anesthetics—sevoflurane, isoflurane, halothane, desflurane and propofol—with a focus on how each agent influences immune cell behavior relevant to infection risk and recovery. A summary of studies discussed in this chapter can be found in Table 1.

### 3.1. Sevoflurane

Sevoflurane is a halogenated volatile anesthetic widely employed for both induction and maintenance of anesthesia in adult and pediatric patients undergoing inpatient and outpatient procedures [23]. Its low blood–gas partition coefficient enables rapid emergence and early recovery compared to agents such as isoflurane and propofol [24].

At the cellular level, one key mechanism responsible for sevoflurane’s immunomodulatory effects involves suppression of the Activator Protein-1 (AP-1) transcription complex. AP-1, composed of c-Jun and c-Fos homo- or heterodimers, regulates the expression of numerous pro-inflammatory genes and is activated via intracellular signaling pathways such as p38 mitogen activated protein kinase (MAPK) [10,25]. In an in vitro model using phorbol myristate acetate (PMA)-stimulated Jurkat T lymphocytes, AP-1 activation was shown to induce nuclear transcription of pro-inflammatory cytokines like IL-2 and TNF-α [26]. Sevoflurane’s interference with AP-1 activation may therefore attenuate inflammation by limiting nuclear transcription of these mediators. While such effects may be protective against tissue injury from excessive inflammation, they may also reduce host immune responses, potentially increasing vulnerability to postoperative infections.

Additionally, several in vitro models have demonstrated that sevoflurane directly impairs key components of innate immune surveillance. Natural killer (NK) cells, particularly the CD56^dim^CD56^bright^ cytotoxic subset, are suppressed by sevoflurane, including reduced secretion of granzyme B—a serine protease essential for the lysis of target cells [11]. Furthermore, sevoflurane downregulates the surface expression of stress-induced ligands major histocompatibility complex (MHC) class I polypeptide-related protein A and B (MICA and MICB) on tumor cells, thereby diminishing recognition by the activating receptor natural killer group 2, member D (NKG2D), which is expressed on NK and CD8^+^ T cells [12,27]. This reduced interaction weakens immune surveillance and cytotoxic responses against transformed or infected cells. Sevoflurane has also been shown to attenuate neutrophil activation following lipopolysaccharide (LPS) stimulation, suppressing the production of elastase and myeloperoxidase—key components of neutrophil antimicrobial activity [13].

Collectively, these findings indicate that while sevoflurane’s anti-inflammatory properties may mitigate tissue damage, they also impair multiple layers of innate immunity, potentially increasing the risk of postoperative infections and facilitating tumor immune evasion.

### 3.2. Isoflurane

Isoflurane is another halogenated volatile anesthetic that has been widely used in clinical practice for decades. Although isoflurane has a higher blood–gas partition coefficient, resulting in slower induction and emergence, it continues to be favored for its affordability and clinical reliability.

Beyond its anesthetic properties, emerging evidence suggests that isoflurane also exerts potent immunomodulatory effects at the cellular level, particularly at sub-anesthetic concentrations. Huang et al. demonstrated that exposure to 0.5 MAC isoflurane in 60% oxygen significantly decreased the inflammatory response in both animal sepsis models and LPS-stimulated human peripheral blood mononuclear cells (PBMCs) [14]. Specifically, isoflurane exposure led to a reduction in the secretion of TNF-α and IL-1β, accompanied by suppressed NF-κB activation, as evidenced by reduced nuclear translocation of the p65 subunit and decreased phosphorylation of IκB kinase (IKK)-β in PBMCs [14].

These immunosuppressive effects of isoflurane are further supported by the findings of Wang et al., who investigated its role in zymosan-induced inflammation in murine Kupffer cells [15]. The study revealed that sub-anesthetic concentrations of isoflurane significantly reduced the production of pro-inflammatory cytokines, including TNF-α, IL-1β, and IL-6, by inhibiting reactive oxygen species (ROS)-mediated activation of the p38 MAPK and NF-κB signaling pathways. Isoflurane was shown to decrease p38 phosphorylation, reduce IκBα degradation, and limit NF-κB nuclear translocation, ultimately impairing the transcription of downstream inflammatory mediators.

Together, these findings suggest that isoflurane plays a dual role in the perioperative setting—not only as a reliable anesthetic agent but also as a modulator of immune responses. By dampening cytokine release and interfering with key inflammatory pathways like NF-κB and MAPK, isoflurane may play a protective role in reducing perioperative inflammation, limiting organ damage, and lowering infection risk, particularly in vulnerable or critically ill patients.

### 3.3. Halothane

Halothane, one of the earliest halogenated volatile anesthetics introduced into clinical practice, played a transformative role in modern anesthesia by offering a potent, non-flammable alternative to ether and chloroform [28]. Despite its historical significance, halothane is associated with serious hepatotoxic effects. Upon hepatic metabolism, it produces reactive intermediates that can bind to liver proteins, forming neoantigens that trigger immune-mediated liver injury. This can lead to halothane-induced hepatitis—a rare but potentially life-threatening condition. The risk is significantly elevated with repeated exposures and is more prevalent among older adults, females, individuals with obesity, or those taking enzyme-inducing medications [29]. While mild cases may resolve spontaneously, severe cases can progress to acute liver failure. Additionally, halothane’s high blood–gas partition coefficient contributes to a slower onset and delayed emergence from anesthesia [30]. In contrast to sevoflurane, which enables rapid recovery, and isoflurane, valued for its cardiovascular stability, halothane is less suitable for contemporary fast-track surgical protocols where rapid turnover is essential [30].

Nonetheless, halothane continues to be of scientific interest, particularly in immunology, where studies have shown that it can alter immune cell function and cytokine production, potentially contributing to perioperative immunosuppression. As with other volatile agents, its immunomodulatory properties appear to be dose- and context-dependent, raising important questions about how different anesthetics influence host defenses and postoperative outcomes. Although halothane is less commonly used today, several in vitro and ex vivo studies have highlighted its significant effects on immune and oxidative stress responses. For example, in a biopsy-based model, halothane exposure led to rapid depletion of key hepatic antioxidants—including reduced glutathione (GSH) and vitamin E—alongside increased levels of malondialdehyde (MDA), a marker of lipid peroxidation, even under normoxic conditions [16]. Additionally, halothane inhibited hepatic superoxide dismutase (SOD) activity, suggesting a direct suppression of endogenous antioxidant defenses and increased susceptibility to oxidative injury.

Beyond oxidative stress, halothane-induced liver injury also involves immune-mediated mechanisms. Cheng et al. showed that natural killer T (NKT) cells play a key role in this process [17]. Mice lacking NKT cells (CD1d^−^/^−^) were resistant to liver damage, showing lower ALT levels, less necrosis, and reduced neutrophil infiltration [17]. This protection was not due to impaired halothane metabolism, as protein adducts and CYP2E1 expression were unaffected. Instead, NKT cells likely promote neutrophil recruitment via cytokines like IL-17. Halothane thus remains a relevant agent in experimental research, offering important insight into the interplay between anesthetic exposure, immune activation, and liver injury.

### 3.4. Desflurane

Among halogenated volatile anesthetics, desflurane is unique in being fully fluorinated, which renders it highly resistant to oxidative metabolism and eliminates the risk of defluorination-related nephrotoxicity observed with agents like sevoflurane and enflurane [31]. However, its pungency and airway irritant properties prohibit its use for inhalational induction, limiting its application to maintenance after intravenous induction. Rapid increases above 1 MAC may cause sympathetic stimulation—presenting as tachycardia and hypertension—and should be used cautiously in hemodynamically unstable patients. Desflurane also poses a rare but serious risk of carbon monoxide formation when used with desiccated CO_2_ absorbents, highlighting the need for fresh gas flow and regular absorbent replacement. Like other halogenated agents, desflurane is a known trigger for malignant hyperthermia and may increase intracranial pressure, limiting its use in neurosurgical patients. Its delivery also requires a heated, pressurized vaporizer, adding cost and limiting availability in resource-constrained settings [31].

Although desflurane is widely used in clinical anesthesia, its immunological profile remains significantly less explored than that of sevoflurane and isoflurane. Both of the latter agents have demonstrated immunosuppressive effects, including the inhibition of transcription factors such as NF-κB, AP-1, and nuclear factor of activated T-cells (NFAT), as well as activation of stress kinases like p38 MAPK [10,18]. In contrast, desflurane exhibits notably different behavior. In vitro studies using PMA/ionomycin-stimulated Jurkat T cells have shown that desflurane does not suppress the DNA-binding activity of NF-κB, AP-1, NFAT, or SP-1, nor does it activate the p38 MAPK pathway or induce apoptosis [10,18]. While this might suggest that desflurane preserves adaptive immune signaling, its effect on innate immunity, particularly in the context of infection, remains poorly defined. This is especially relevant given that postoperative infection is often driven by early neutrophil recruitment and extracellular matrix remodeling, processes heavily influenced by matrix metalloproteinase-9 (MMP-9). Although one study demonstrated that desflurane reduces cancer cell invasiveness by downregulating the Akt–MMP-9 signaling axis, it is unclear whether this effect translates into impaired neutrophil function or delayed tissue repair during postoperative infection [19].

While numerous studies have investigated the immunosuppressive and anti-inflammatory properties of sevoflurane, isoflurane, and propofol—particularly their effects on cytokine production, leukocyte activation, and transcription factor signaling—the literature surrounding desflurane remains sparse. This limited evidence makes it unclear whether desflurane acts similarly or differently in modulating immune responses, particularly during the perioperative period when immune function is critical for infection control and recovery. This gap in knowledge supports the need for further investigation using pathogen-relevant in vitro models to determine whether desflurane’s immunomodulatory profile may contribute to increased susceptibility to postoperative infection.

### 3.5. Propofol

Propofol is a widely used intravenous anesthetic known for its rapid onset, short duration of action, and favorable recovery profile, making it ideal for both induction and maintenance of anesthesia [32]. Unlike volatile agents, propofol is administered via continuous infusion and is especially favored in outpatient surgeries and intensive care settings due to its smooth emergence and antiemetic properties. Its mechanism of action primarily involves potentiation of GABA(A) receptor activity, leading to enhanced inhibitory neurotransmission in the central nervous system [33]. However, there is increasing evidence that propofol also plays a significant role in immune modulation.

In vitro studies have shown that propofol can directly influence both innate and adaptive immune cell function. Kochiyama et al. demonstrated that propofol significantly suppressed the production of pro-inflammatory cytokines IL-1β and IL-6 in LPS-stimulated human M1 macrophages, indicating direct inhibition of innate immune activation at the cellular level [20]. Focusing on adaptive immunity, Yamamoto et al. reported that propofol impairs T cell effector function by downregulating glycolytic metabolism in activated CD8^+^ T cells, leading to reduced cytokine secretion (IL-2, IFN-γ, TNF-α) and diminished cytotoxic activity in vitro [21]. CD4^+^ T helper (Th) cells are central regulators of adaptive immunity, directing B cell antibody production, CD8^+^ T cell responses, macrophage activation, and immunological memory. During stimulation of their receptors in specific cytokine milieus, naïve CD4^+^ T cells differentiate into subsets such as Th1, Th2, Th17, or Foxp3^+^ regulatory T cells, each defined by their cytokine profiles and effector roles [34]. Propofol’s effects extended to impaired differentiation of Th1, Th2, and Th17 cells, along with enhanced Foxp3^+^ Treg skewing under Th17 conditions. Additionally, Hiraoka et al. showed that propofol suppresses T cell–dependent antibody responses by directly inhibiting IL-2–driven proliferation and IL-7–mediated survival of naïve CD4^+^ T cells in culture [22].

Together, these in vitro findings provide strong mechanistic evidence that propofol actively modulates both innate and adaptive immune functions, independent of surgical stress or other perioperative variables.

## 4. In Vivo Evidence

This chapter reviews key animal-based studies exploring the physiological and immunological effects of anesthetics such as sevoflurane, isoflurane, propofol, ketamine, and regional techniques. Notably, volatile agents like sevoflurane have been shown to enhance survival in septic models by supporting innate mechanisms and modulating inflammatory signaling [35], while propofol maintains homeostasis without promoting excessive immune activation [36]. Regional techniques cause minimal disruption and support healing [6]. The integration of these findings emphasizes the active role of anesthetics in biological recovery [37]. By tracing the immune impacts of anesthetics in animal models, this chapter aims to provide a nuanced perspective on the evolving role of anesthesia in shaping postoperative outcomes. Table 2 offers a comprehensive overview of studies examined in this chapter.

### 4.1. Wound Healing and Tissue Regeneration Models

A central theme in the recent literature is how anesthetics affect wound healing and tissue regeneration. Choi et al. examined wound healing in rats administered with either sevoflurane or propofol for varying durations [38]. The study revealed that short-term sevoflurane administration improved local blood flow and slightly improved would healing, while longer exposures (4–8 h) significantly delayed wound closure and impaired tissue regeneration. Conversely, propofol maintained consistent perfusion and healing regardless of exposure time. These findings challenge the idea that sevoflurane universally benefits immune system recovery and highlight the importance of the dose and duration of anesthesia.

### 4.2. Sepsis Models

To explore how anesthetics affect host survival and immune response during sepsis, Schläpfer et al. used a rat model of cecal ligation and puncture (CLP) to simulate polymicrobial infection and compare outcomes between propofol and volatile agents [39]. Rats sedated with propofol showed significantly higher mortality and morbidity, along with increased pulmonary vascular permeability, elevated IL-6 levels, and greater endothelial dysfunction. These findings suggest that propofol may worsen immune outcomes during sepsis by disrupting both vascular and immune stability.

In a complementary approach, Olivera et al. collected immune cells from CLP-induced septic rats and exposed them ex vivo to either sevoflurane or propofol [40]. The results revealed that sevoflurane treatment upregulated anti-inflammatory cytokines (IL-10m TGF-β), suppressed pro-inflammatory gene expression (IL-1β, IL-6), and enhanced neutrophil migration and phagocytosis—indicating direct immune activation. In contrast, propofol had minimal impact on these immune markers and uniquely decreased epithelial surfactant protein B, potentially compromising alveolar defense.

Building on Olivera et al.’s mechanistic findings and contrasting Schläpfer et al., a 2021 study performed by Liu et al., extended this work into a complete in vivo setting [39,40,41]. In this study, mice subjected to CLP and subsequent sevoflurane anesthesia showed significantly improved seven-day survival, lower systemic bacterial load, and significantly lower pro-inflammatory cytokine levels compared to non-anesthetized septic controls [41]. Furthermore, sevoflurane also mitigated organ damage, suggesting that its benefits extend beyond immune modulation. These findings build on earlier studies demonstrating that sevoflurane not only improves cellular immune activity but also improves overall survival during infection.

Overall, these studies suggest that volatile anesthetics like sevoflurane can actively shape the immune response, help the body fight infection, and improve survival during systemic inflammation. Conversely, propofol appears to be less effective in supporting the adaptive immune system under these conditions. However, as noted by Choi et al., long-term exposure to volatile agents, despite reducing inflammation, may still delay tissue healing, highlighting the need to balance immune modulation with recovery time [38]. Altogether, these in vivo studies support the idea that volatile anesthetics like sevoflurane influence host immunity in ways which may improve outcomes in the postoperative phase.

### 4.3. Viral and Bacterial Infection Models

These findings gain a meaningful context when considered alongside studies using disease models without surgery. Penna et al. explored how anesthetics influence the immune response to viral infection using an influenza A mouse model [42]. Mice anesthetized with ketamine/xylazine had higher pulmonary viral loads, worsened lung histopathology, and reduced levels of immune injury markers compared to those given halothane. This demonstrated that ketamine-based anesthesia can attenuate antiviral defenses and contributed to more severe disease even in the absence of surgery [42].

Building on this perspective, Woodrow et al., investigated immune response to anesthesia in a non-infectious, non-surgical equine model [43]. In this study, horses underwent two hours of general anesthesia, after which bronchoalveolar lavage samples were collected. These samples revealed reduced TNF-α and IL-6 production in response to LPS challenge, suggesting that anesthetic agents along could reduce immune responsiveness, particularly within pulmonary tissues. These findings demonstrate that even in the absence of infection or tissue damage, general anesthesia seemed to lower the ability of the lungs to respond to inflammatory signals.

To explore how anesthetics may influence infection control more directly, Visvabhrathy et al. investigated the effect of propofol on postoperative bacterial infection using Listeria monocytogenes [44]. Mice anesthetized with a single clinical dose of propofol prior to infection exhibited significantly higher bacterial loads in the spleen and liver, increased histopathologic tissue damage, and elevated mortality compared to non-anesthetized controls. These effects occurred in the absence of any surgical procedure, suggesting that propofol alone may weaken the body’s ability to fight off infection. Instead of clearing bacterial from tissues such as the liver and spleen, propofol seemed to worsen bacterial spread and organ damage, raising concerns about its role in infection control during the postoperative phase.

In summary, these in vivo studies demonstrate that anesthetic choice can significantly shape postoperative outcome. Volatile agents like sevoflurane show promise in supporting immune defense, while agents like propofol may carry greater risks in inflammatory settings. Altogether, these findings highlight the importance of considering immune effects during the perioperative period when selecting anesthetics, particularly in patients at risk of infection or delayed recovery.

## 5. Clinical Evidence

Understanding the clinical implications of anesthetic agents on immune system modulation is crucial for improving perioperative outcomes, particularly with respect to postoperative infections. This chapter examines current clinical evidence on the impact of anesthetic choice on postoperative infection rates, wound healing, and the severity of complications across various surgical populations. It includes findings from both retrospective cohort studies and randomized controlled trials, allowing for the inclusion of a range of adult and pediatric populations. By comparing the differential effects of intravenous and volatile anesthetics on immune function and clinical recovery, this chapter aims to inform clinical decisions to improve patients’ immune outcomes after surgery. The various clinical studies discussed in this chapter are outlined in Table 3.

### 5.1. Retrospective Studies in Adult Populations

Retrospective studies are a valuable tool to identify trends in clinical outcomes across large populations. A retrospective study conducted by Weiss et al., investigated postoperative infection rates in 1462 patients undergoing cardiac surgery who received either etomidate or propofol as induction anesthetics [45]. After matching patients from both anesthetic groups based on factors such as surgical urgency and disease severity, they found that hospital-acquired pneumonia was significantly more common in the etomidate group (18.6%) compared to the propofol group (14.0%). Furthermore, they also quantified sepsis rates among groups, reporting that sepsis was more frequent when etomidate was used (11.5%) compared to propofol (8.2%), although this difference did not reach statistical significance. The authors suggest that etomidate’s known effects on adrenal suppression may play a critical role in the differences observed in infection rates.

A similar focus on pulmonary complications was examined by Zhang and Wang in a retrospective cohort study, which explored the differences between propofol and the commonly used volatile anesthetic sevoflurane [46]. The study included 1659 patients who underwent esophagectomy for cancer, and after propensity score matching, 78 patients from each group were included. The incidence of postoperative pneumonia was 7.7% in the sevoflurane group and 6.4% in the propofol group; however, the difference was not statistically significant. Alcohol use, surgical procedure, and surgeon experience were identified as independent predictors of postoperative pneumonia. This suggests that while anesthetic choice may play a role, other perioperative factors can also influence postoperative outcomes. 

Aside from pulmonary outcomes of surgery, many retrospective studies have also investigated the impact of anesthetic choice on SSIs. Hu et al. conducted a retrospective analysis comparing anesthesia with dexmedetomidine (DEX) and propofol in patients undergoing coronary artery bypass graft surgery [47]. Patients anesthetized with DEX had significantly fewer pulmonary complications (7.8% vs. 13.3% with propofol, *p* < 0.01) and had a lower rate of wound infection and dehiscence (2.5% vs. 6.6%, *p* < 0.001). Additionally, patients in the DEX groups spent less time on mechanical ventilation and had shorter ICU and hospital stays. Although 30-day mortality was similar between groups, the lower complication and infection rates suggest that DEX may offer some protection in postoperative settings. These findings highlight the idea that immune-modulating effects of anesthetics can differ between volatile and intravenous agents, but among different intravenous agents themselves. 

Further supporting the role of anesthetics in the development of surgical site infections is a study conducted by Koo et al., which investigated the difference in surgical site infection rates in colorectal surgery patients anesthetized with either volatile gases or propofol [48]. After matching propensity scores of 1934 patients, 390 patients were included in each group for the final analysis. It was reported that SSI occurred more frequently in the volatile group (2.6%), consisting of patients anesthetized with either sevoflurane or desflurane, compared to the propofol group (0.5%), with an odds ratio of 5.0 (95% CI: 1.1–22.8, *p* = 0.039). Furthermore, they reported that postoperative inflammatory markers, such as C-reactive protein and white blood cell count, were significantly higher in the volatile group than the propofol group. These findings suggest that the anti-inflammatory and antioxidant properties of propofol may contribute to reduced SSI rates in specific surgical contexts. 

In contrast to the findings by Koo et al., a retrospective study conducted by Shimizu et al. reported higher surgical site infection rates among patients who received propofol compared to those who received sevoflurane after elective open gastrointestinal surgery [48,49]. Using a propensity score model, researchers matched 84 pairs of patients from each anesthetic group. They reported that SSIs occurred in 7.1% of patients who received sevoflurane and 16.7% of those who received propofol. Additionally, the standardized infection ratio (SIR) is significantly lower in the sevoflurane group (1.89) compared to the propofol group (4.78, *p* = 0.02). The authors suggest that differences in oxidative activity between anesthetics may partially explain these findings. Sevoflurane has been shown to increase reactive oxygen species (ROS) production, while propofol has demonstrated antioxidant properties. These results further support the notion that immunological outcomes vary based on the location and complexity of the surgical procedure, as well as patient-specific factors. 

Moreover, Yamamoto et al. conducted a retrospective study evaluating perioperative and anesthetic risk factors for SSI in 326 patients undergoing pancreaticoduodectomy [50]. The overall SSI rate in this high-risk surgical population was 18.4%. A multivariable analysis revealed that the use of desflurane as a maintenance anesthetic was associated with significantly lower SSI risk than sevoflurane (OR = 0.503; 95% CI, 0.260 = 0.973). Aside from anesthetic choice, significant risk factors for developing an SSI also included prolonged surgery time, cerebrovascular disease, and ischemic heart disease. These findings further emphasize that even within the same class of volatile anesthetic agents, differences in pharmacological properties can have a significant impact on patient outcomes.

Lastly, an extensive retrospective cohort study conducted by Kishimoto et al. compared anesthesia with propofol to sevoflurane in over 21,000 patients undergoing total knee arthroplasty [51]. The study examined early joint infection rates and reported no significant difference between the groups (propofol: 1.3% vs. sevoflurane: 1.7%). Given the low infection rates in both anesthetic groups, it was concluded that the choice of anesthetic does not significantly impact the outcome in the context of this total knee arthroplasty study.

### 5.2. Retrospective Studies in Pediatric Populations

Compared to adult populations, there is limited clinical data investigating how anesthetic choice influences postoperative infections in pediatric patients. Shibamura-Fujiogi et al. conducted a retrospective study of 621 pediatric patients undergoing elective intestinal surgery to investigate whether anesthetic dose influenced the risk of developing an SSI [52]. After propensity score matching, patients were compared based on whether they received high or low doses of sevoflurane for anesthesia maintenance (median dose = 272.5%∗min). They reported that the incidence of SSIs was significantly higher in the high-dose group (9.8%) compared to the low-dose group (3.9%, *p* = 0.019). This difference remained significant after adjusting for blood transfusion and duration of anesthesia (OR 2.58, 95%; Cl: 1.1–6.04). While further studies are required to investigate the underlying mechanisms and further explore these results, this study demonstrates that anesthetic dosing may have an impact on postoperative outcomes, not just the choice of anesthetic agent used.

### 5.3. Randomized Controlled Trials

Randomized controlled trials (RCTs) are critical for determining causality between anesthetic choice and postoperative infection risks. In a randomized clinical trial conducted by Zhang et al., volatile anesthetics sevoflurane and isoflurane were compared with propofol in 553 patients undergoing a minimally invasive esophagectomy [54]. It was reported that patients who received volatile anesthesia had a significantly lower incidence of postoperative pulmonary complications (36.5%) compared to those who received propofol (47.5%, *p* = 0.013). Among the pulmonary complications, the most commonly reported was respiratory infection, which occurred less frequently in the volatile group (30.0%) compared to the propofol group (38.8%, *p* = 0.038). In addition to the overall rate of complications, the severity of complications was also lower in the volatile group, with fewer patients experiencing severe pulmonary complications (grade ≥ 3 on the postoperative pulmonary complication severity score, ranging from 0 to 5) (33.8% vs. 44.8%, *p* = 0.012). The authors suggest that volatile anesthetics may reduce pulmonary complications through their anti-inflammatory effects, characterized by higher IL-10 levels and lower IL-6 and TNF-α levels.

In addition to individual RCTs, Alhayyan et al. conducted a meta-analysis of over 60 RCTs to assess the influence of different anesthetics on the postoperative inflammatory response and potential complications [53]. In this study, researchers specifically examined C-reactive protein (CRP) and IL-6 levels, which are key markers of systemic inflammation. Across studies involving various surgical procedures, it was reported that total intravenous anesthesia (TIVA) with propofol significantly reduced CRP levels (*p* = 0.04). Conversely, other anesthetic techniques, such as general volatile gases, regional, or combined anesthesia, did not show significant reductions in CRP or IL-6 levels. These findings support the idea that propofol-based TIVA may have anti-inflammatory properties that lessen postoperative infection risk and improve patient outcomes.

## 6. Limitations

Anesthetic drugs, as discussed above, have significant impacts on immune function, but it is difficult to isolate their effects from other factors. Surgery itself alters immune responses, and additional complications, such as sepsis, viral, or bacterial infections, can further contribute to the effects observed with anesthetic agents. Furthermore, patient characteristics, including age, sex, pre-existing immune conditions, transplant status, and medications, can play a role in shaping patient outcomes. Additionally, aspects of anesthetic management as well as hypotensive episodes, reduced blood flow, blood transfusions, hypothermia, timing and dosing of antibiotics and sterile workplace environment can influence the occurrence of postoperative infections. These possible confounding variables make it challenging to determine which changes are caused by the drugs themselves and which are a result of the patient’s treatment or condition. Most clinical evidence originates from retrospective studies, which often have many unadjusted confounding variables, further complicating interpretation. These limitations make it challenging to draw conclusions about the direct effects of anesthetics on immune function and postoperative infection risk, underscoring the need for further studies, particularly randomized controlled trials, to establish causation.

## 7. Conclusions

In summary, the research presented here demonstrates the ability of anesthetics to play a critical role in immune responses and recovery after surgery. In vitro studies show that volatile agents such as sevoflurane and isoflurane can dampen inflammatory signaling pathways, which may be beneficial in reducing tissue damage. However, this suppression may impair the body’s ability to mount an effective immune response to infection. Propofol is often described as anti-inflammatory, but recent evidence suggests it may suppress critical components of the adaptive immune system, such as T cell activation and antibody production. Consequently, host defense and pathogen clearance may be weakened in the postoperative setting.

Animal models have supported these results, reporting that volatile anesthetics like sevoflurane have been shown to improve survival in sepsis and enhance critical immune functions such as phagocytosis and cytokine regulation. Propofol, on the other hand, has been linked to worse outcomes, including higher bacterial loan and reduced immune function.

Clinically, current evidence from retrospective studies and randomized controlled trials show mixed results, depending on surgery type, anesthetic dosing, and unadjusted patient-specific confounders. These findings highlight the need for future research to focus on randomized controlled trials, allowing causation to be established between anesthetics and postoperative infection risk. Better understanding these effects will be critical to better guide anesthetic choices that support both immune protection and surgical success.

## Figures and Tables

**Table 1:** Summary of in vitro studies examining the impact of anesthetics on immune modulation and infection-related outcomes.

Anesthetic	Model	Methods	Results	Reference
Sevoflurane	Primary human CD3+ lymphocytes	Electrophoretic mobility shift assay (AP-1 DNA binding); Western blot (MAPK phosphorylation); Reporter gene assay (AP-1 activity); ELISA (cytokine release).	Sevoflurane is a specific inhibitor of AP-1 activation and transcriptional activity via altered p38 MAPK phosphorylation.	[10]
Isoflurane			Isoflurane showed no AP-1 inhibition.	
Desflurane			Desflurane showed no AP-1 inhibition.	
Sevoflurane	Human NK cell subpopulations (PBMC-derived)	Flow cytometry (NK subsets: CD56^dimCD16^bright, etc.), perforin/GrzB expression.	Reduced cytotoxic NK subset (CD56^dimCD16^bright) by −16.1%; Reduced granzyme B (GrzB) secretion.	[11]
Propofol			Reduced cytotoxic NK subset (CD56^dimCD16^bright) by −3.58%; No difference in GrzB secretion compared to sevoflurane.	
Sevoflurane	Human breast cancer cell lines (MCF-7, MDA-MB-453, HCC-70) co-cultured with NK cells	qPCR & flow cytometry (NKG2D ligands); Western blot (MMP-1/2); ELISA (soluble ligands); Cytotoxicity assays.	Downregulated surface expression of MICA and MICB—decreasing identification from NK cells and CD8+ via NKG2D.	[12]
Sevoflurane	Human PMNs (whole blood)	In vitro exposure (2.3–4.6%); chemiluminescence (ROS); ELISA (MPO, elastase); SIEFED (active MPO).	Inhibited neutrophil activation after LPS stimulation (decrease in MPO and elastase); No significant change in ROS.	[13]
Isoflurane	Mouse RAW264.7 macrophages and human PBMCs (LPS-stimulated)	Western blot & immunohistochemistry (NF-κB: p-IKKβ, p-IκBβ, p-p65); Cytokine assays (TNF-α, IL-1β, IL-6, HMGB1).	Reduction in the secretion of TNF-α and IL-1β; NF-κB activation suppression.	[14]
Isoflurane	Murine Kupffer cells (zymosan-stimulated)	Western blot (COX-2, p38 MAPK); Radioimmunoassay (PGE_2_); ELISA (cytokines, chemokines); EMSA (NF-κB).	Reduction in TNF-α, IL-1β, and IL-6; Suppressed ROS-induced activation of p38 and NF-κB signaling pathways; Decreased p38 phosphorylation, reduced IκBα degradation, and limited NF-κB nuclear translocation.	[15]
Halothane	Dogs (liver & plasma) biopsy-based model	1% halothane under normoxia or hypoxia; serial liver biopsies & plasma at 0–60 min; assays: GSH, vitamin E, vitamin C, MDA, SOD.	↓ GSH, ↓ Vit E, ↑ MDA (lipid peroxidation), ↓ hepatic SOD *. Effects most pronounced under hypoxia, but depletion evident even in normoxia.	[16]
Halothane	Hepatocytes & immune cells (murine HILI model)	Hepatocyte stress assays (oxidative stress: GSH, MDA, SOD); WT vs. CD1d^−^/^−^ mice (NKT deficiency).	NKT-deficient mice were resistant, showing NKT-dependent neutrophil recruitment following halothane-induced liver injury.	[17]
Sevoflurane	Jurkat T-cells	2.5–8% exposure; Western blot for p38, ASK1, MKK3/6, ATF-2; caspase-3 activity assay; Annexin-V/PI flow cytometry	Inhibition of NF-κB, AP-1, and NFAT; Activation of p38, MAPK.	[18]
Isoflurane				
Desflurane			No suppression of DNA-binding activity of NF-κB, AP-1, NFAT, or SP-1; No p38 MAPK pathway activation; No apoptosis induction.	
Desflurane	Human neutrophils ± MC-38 colon cancer cells	ELISA & zymography (MMP-9); Flow cytometry (CXCR2, ERK1/2); Matrigel invasion assay	Downregulated the IL-8 induced Akt–MMP-9 signaling axis.	[19]
Sevoflurane			Downregulated IL-8 induced Akt–MMP-9 signaling axis.	
Propofol	Human macrophages (M1/M2 polarization)	Polarization assays; ELISA (IL-6, IL-1β, TNF-α, IL-10, TGF-β); qPCR (CD206); siRNA (Nrf2); Immunofluorescence (Nrf2).	Suppression of IL-1β and IL-6 in LPS-stimulated M1 macrophages.	[20]
Propofol	Human/murine CD8^+^ T cells	Metabolic assays (glycolysis); Flow cytometry (effector differentiation); Cytokine assays; Cytotoxicity assays.	Suppressed glycolysis in activated CD8+ T cells; Reduced cytokine release (IL-2, IFN-γ, TNF-α); Reduced cytotoxic antitumor activity; Impaired differentiation of T cells.	[21]
Propofol	Murine CD4^+^ T cells	Proliferation (IL-2); Survival (IL-7) assays.	Suppression of T cell–dependent antibody responses; Inhibition of IL-7-mediated survival of naïve CD4+ T cells.	[22]

* Arrows indicate a direction of change such that ↓ indicates a decrease and ↑ indicates an increase.

**Table 2:** Summary of in vivo experimental models and outcomes exploring the impact of anesthetics on immune modulation and infection-related outcomes.

Anesthetic	Model	Methods	Result	Reference
Sevoflurane vs. Propofol	Rats (wound healing model)	Tissue perfusion, wound closure rate	Sevoflurane short-term increased perfusion; long-term delayed healing.	[38]
Propofol	Rats (CLP sepsis model)	IL-6 levels, vascular permeability, survival	Increased mortality; increased IL-6, vascular dysfunction	[39]
Sevoflurane vs. Propofol	Septic Rats (immune cells ex vivo)	IL-10, TGF-β release, neutrophil phagocytosis	Sevoflurane increased anti-inflammatory cytokines & phagocytosis	[40]
Sevoflurane	Septic mice (CLP model)	Survival; bacterial load, IL-1β, TNF-α, IL-6 levels	Increased survival; Decreased bacterial load and cytokines.	[41]
Ketamine/Xylazine vs. Halothane	Mice (influenza A infection)	Viral load; lung histopathology	Ketamine increased viral load and worsened pathology.	[42]
Volatile Anesthesia	Horses (BAL samples)	LPS stimulation; TNF-α, IL-6 production.	Decreased pulmonary cytokine response.	[43]
Propofol	Mice (Listeria infection)	Bacterial burden; histopathology; survival	Increased bacterial load and mortality.	[44]

**Table 3:** Summary of clinical studies examining the impact of anesthetic choice on immune modulation and postoperative infection outcomes.

Anesthetic	Patient Population	Outcome	Result	Reference
Etomidate vs. Propofol	Cardiac surgery patients	Pneumonia, sepsis incidence	Etomidate increased pneumonia and sepsis	[45]
Sevoflurane vs. Propofol	Esophagectomy patients	Pneumonia incidence	No significant difference	[46]
Dexmedetomidine vs. Propofol	Coronary artery bypass graft surgery patients	Pulmonary and wound infection rates.	DEX reduces infection rates	[47]
Volatile vs. Propofol	Colorectal surgery patients	SSI incidence	SSI higher with volatiles vs. propofol	[48]
Sevoflurane vs. Propofol	Gastrointestinal surgery patients	SSI incidence	SSI higher with Propofol vs. sevoflurane	[49]
Sevoflurane vs. Desflurane	Pancreaticoduodenectomy patients	SSI incidence	Lower SSI with desflurane	[50]
Sevoflurane vs. Propofol	Total knee arthroplasty patients	Joint infection incidence	No significant difference	[51]
Sevoflurane (dose effect)	Pediatric intestinal surgery patients	SSI incidence	High dose sevoflurane—higher SSI	[52]
Volatile vs. Propofol	Esophagectomy patients	Pulmonary infection outcomes	Volatile decreased pulmonary infections (higher IL-10, lower IL-6 and TNF-α.	[53]
Multiple anesthetics	Various surgical procedures	C-reactive protein, IL-6 levels	Propofol total intravenous anesthesia decreased C-reactive protein levels, volatiles had no effect.	[54]

## Data Availability

No new data were created or analyzed in this study. Data sharing is not applicable to this article.
